# Supplementary material for: A CD4+ T cell reference map delineates subtype-specific adaptation during acute and chronic viral infections
Source: eLife. 2022 Jul 13;11:e76339. doi: 10.7554/eLife.76339 (PMC9323004; doi:10.7554/eLife.76339)
Supplement: Transparent reporting form [file elife-76339-transrepform1.docx]

***eLife’s* transparent reporting form**

We encourage authors to provide detailed information *within their submission* to facilitate the interpretation and replication of experiments. Authors can upload supporting documentation to indicate the use of appropriate reporting guidelines for health-related research (see [EQUATOR Network](http://www.equator-network.org/%20)), life science research (see the [BioSharing Information Resource](https://biosharing.org/)), or the [ARRIVE guidelines](http://www.plosbiology.org/article/info:doi/10.1371/journal.pbio.1000412) for reporting work involving animal research. Where applicable, authors should refer to any relevant reporting standards documents in this form.

If you have any questions, please consult our Journal Policies and/or contact us: [editorial@elifesciences.org](mailto:editorial@elifesciences.org).

**Sample-size estimation**

- You should state whether an appropriate sample size was computed when the study was being designed
- You should state the statistical method of sample size computation and any required assumptions
- If no explicit power analysis was used, you should describe how you decided what sample (replicate) size (number) to use

Please outline where this information can be found within the submission (e.g., sections or figure legends), or explain why this information doesn’t apply to your submission:

Empiric sample-size estimation was used for genomic datasets with at least 2 replicates for each timepoint. For in vivo validations, at least 5 animals per groups were utilized for Fig 1, Fig 3 E-F, Fig S1, Fig S3 B-D.

**Replicates**

- You should report how often each experiment was performed
- You should include a definition of biological versus technical replication
- The data obtained should be provided and sufficient information should be provided to indicate the number of independent biological and/or technical replicates
- If you encountered any outliers, you should describe how these were handled
- Criteria for exclusion/inclusion of data should be clearly stated
- High-throughput sequence data should be uploaded before submission, with a private link for reviewers provided (these are available from both GEO and ArrayExpress)

Please outline where this information can be found within the submission (e.g., sections or figure legends), or explain why this information doesn’t apply to your submission:

All datasets and timepoints have at least 2 biological (independent animals) and/or technical replicates (independent scRNAseq capture and sequencing) as described in Data Supplemental 1.

All in vivo validations were performed at least in 2 independent experiments with 3-5 animals per groups in each experiment.

Sequence data are deposited in the NCBI Gene Expression Omnibus under accession numbers GSE182320 and GSE200635..

**Statistical reporting**

- Statistical analysis methods should be described and justified
- Raw data should be presented in figures whenever informative to do so (typically when N per group is less than 10)
- For each experiment, you should identify the statistical tests used, exact values of N, definitions of center, methods of multiple test correction, and dispersion and precision measures (e.g., mean, median, SD, SEM, confidence intervals; and, for the major substantive results, a measure of effect size (e.g., Pearson's r, Cohen's d)
- Report exact p-values wherever possible alongside the summary statistics and 95% confidence intervals. These should be reported for all key questions and not only when the p-value is less than 0.05.

Please outline where this information can be found within the submission (e.g., sections or figure legends), or explain why this information doesn’t apply to your submission:

Statistical comparisons were done by one-way ANOVA test. P-values are indicated in graphs Fig 3E, F and Fig S3 B-D.

All other statistical methods used to analyze transcriptomic data are available at <https://github.com/carmonalab> (eg <https://github.com/carmonalab/ProjecTILs_CaseStudies/blob/master/CaseStudy_Kunzli2020.Rmd> ).

(For large datasets, or papers with a very large number of statistical tests, you may upload a single table file with tests, Ns, etc., with reference to sections in the manuscript.)

**Group allocation**

- Indicate how samples were allocated into experimental groups (in the case of clinical studies, please specify allocation to treatment method); if randomization was used, please also state if restricted randomization was applied
- Indicate if masking was used during group allocation, data collection and/or data analysis

Please outline where this information can be found within the submission (e.g., sections or figure legends), or explain why this information doesn’t apply to your submission:

Randomization was not applied in the study.

**Additional data files (“source data”)**

- We encourage you to upload relevant additional data files, such as numerical data that are represented as a graph in a figure, or as a summary table
- Where provided, these should be in the most useful format, and they can be uploaded as “Source data” files linked to a main figure or table
- Include model definition files including the full list of parameters used
- Include code used for data analysis (e.g., R, MatLab)
- Avoid stating that data files are “available upon request”

Please indicate the figures or tables for which source data files have been provided:

Sequence data are deposited in the NCBI Gene Expression Omnibus under accession

number GSE182320 and GSE200635. Reference atlas can be downloaded (DOI: 10.6084/m9.figshare.16592693) or accessed via the web portal (<https://spica.unil.ch/refs/viral-CD4-T>). All code sources are available at <https://github.com/carmonalab> (e.g. <https://github.com/carmonalab/ProjecTILs_CaseStudies/blob/master/CaseStudy_Kunzli2020.Rmd> )
